# Celastrol Attenuates Inflammatory and Neuropathic Pain Mediated by Cannabinoid Receptor Type 2

**DOI:** 10.3390/ijms150813637

**Published:** 2014-08-06

**Authors:** Longhe Yang, Yanting Li, Jie Ren, Chenggang Zhu, Jin Fu, Donghai Lin, Yan Qiu

**Affiliations:** 1 Department of Medical Sciences, Medical College, Xiamen University, Xiamen 361006, China; E-Mails: longhe.yang@gmail.com (L.Y.); 24520121153220@stu.xmu.edu.cn (Y.L.); renjie_7912@xmu.edu.cn (J.R.); chenggang.zhu@gmail.com (C.Z.); fujanie@gmail.com (J.F.); 2 The Key Laboratory for Chemical Biology of Fujian Province, College of Chemistry and Chemical Engineering, Xiamen University, Xiamen 361005, China

**Keywords:** celastrol, inflammatory pain, neuropathic pain, cannabinoid receptors

## Abstract

Celastrol, a major active ingredient of Chinese herb *Tripterygium wilfordii* Hook. f. (thunder god vine), has exhibited a broad spectrum of pharmacological activities, including anti-inflammation, anti-cancer and immunosuppression. In the present study, we used animal models of inflammatory pain and neuropathic pain, generated by carrageenan injection and spared nerve injury (SNI), respectively, to evaluate the effect of celastrol and to address the mechanisms underlying pain processing. Intraperitoneal (i.p.) injection of celastrol produced a dose-dependent inhibition of carrageenan-induced edema and allodynia. Real-time PCR analysis showed that celastrol (0.3 mg/kg, i.p.) significantly reduced mRNA expressions of inflammatory cytokines, TNF-α, IL-6, IL-1β, in carrageenan-injected mice. In SNI mice, pain behavior studies showed that celastrol (1 mg/kg, i.p.) effectively prevented the hypersensitivity of mechanical nociceptive response on the third day post-surgery and the seventh day post-surgery. Furthermore, the anti-hyperalgesic effects of celastrol in carrageenan-injected mice and SNI mice were reversed by SR144528 (1 mg/kg, i.p.), a specific cannabinoid receptor-2 (CB_2_) receptor antagonist, but not by SR141716 (1 mg/kg, i.p.), a specific cannabinoid receptor-1 (CB_1_) receptor antagonist. Taken together, our results demonstrate the analgesia effects of celastrol through CB_2_ signaling and propose the potential of exploiting celastrol as a novel candidate for pain relief.

## 1. Introduction

Herb extracts of *Tripterygium Wilfordii* Hook. f. (*T. wilfordii*) have been widely used in China for years as anti-inflammation agents in many chronic inflammatory diseases and autoimmune diseases [1,2]. Celastrol, a major component of *T. wilfordii*, has exerted a profound anti-inflammatory effect on various animal models, including rheumatoid arthritis [3,4], atherosclerosis [5], Alzheimer’s disease [6,7], asthma [8] and systemic lupus erythematosus [9]. However, the mechanisms underlying the anti-inflammatory action of celastrol have not been fully explored.

Endocannabinoids have been discovered as one of the most common endogenous systems engaged in the modulation of inflammation and pain perception [10,11,12]. They comprise two major components, anandamide (AEA) and 2-arachidonoylglycerol (2-AG), along with two cannabinoid receptors, cannabinoid receptor-1 (CB_1_) and cannabinoid receptor-2 (CB_2_), and the enzymes involved in their biosynthesis and hydrolysis [10]. The activation of CB_1_ and CB_2_ signals produces antinociceptive and anti-hyperalgesic effects at peripheral and central levels [13,14]. Although the distribution of the CB_1_ receptor in primary afferent neurons is attributed to the analgesia effect, the activation of the CB_1_ receptor is associated with the central side effects, including ataxia, psychosis and catalepsy [15]. Recent findings suggest that the CB_2_ receptor also contributes to anti-hyperalgesia by inhibiting the release of proinflammatory factors from non-neuronal cells located near nociceptive neuron terminals [16]. CB_2_ is expressed in several types of inflammatory cells and immune cells, and the activation of the peripheral CB_2_ receptor generates an antinociceptive response in inflammatory pain and neuropathic pain [17,18]. Therefore, selective activation of the CB_2_ receptor has potential to reduce pain without inducing the centrally-mediated side effects.

Previous studies showed that targeting a cannabinoid-hydrolyzing enzyme, monoacylglycerol lipase (MGL), attenuated inflammatory pain and neuropathic pain through elevating 2-AG levels, which potently activated the CB_2_ receptor [19,20,21]. As natural herbs are important resources for drug discovery, many compounds from natural herbs have been identified as analgesia agents targeting the endocannabinoid system [22,23]. King* et al.* [24] found natural triterpenoid compounds, celastrol and euphol, exhibiting the potent inhibition of MGL activity with IC_50_ values of 1.6 ± 0.4, 0.31 ± 0.08 μM, respectively. Recently, Dutra *et al.* demonstrated that oral administration of euphol effectively prevented hyperalgesia induced by carrageenan and ligation of the sciatic nerve through the cannabinoid-mediated pathway [25].

In the present study, we assessed the effect of natural compound celastrol and elucidated the mechanisms underlying celastrol’s action in preventing inflammatory and neuropathic pain. Furthermore, we investigated the cytokine status responding to the inflammatory pain and the effects of celastrol on cytokine-mediated nociception. As few drugs are currently available for the treatment of chronic pain, our study provides the evidence that celastrol might be a promising molecule for the management of inflammatory and neuropathic pain.

## 2. Results and Discussion

### 2.1. Celastrol Dose- and Time-Dependently Reduced Carrageenan-Induced Edema and Hyperalgesia

**Figure 1:**
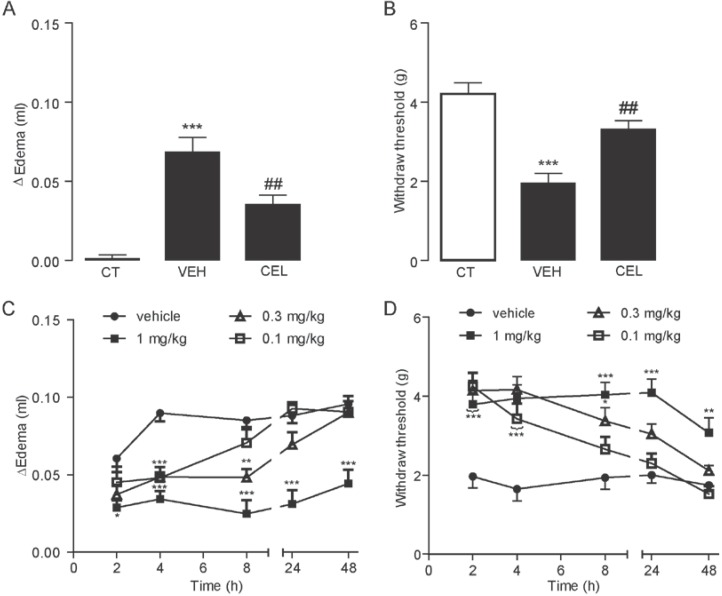
Celastrol reduced edema and hyperalgesia in the carrageenan-induced inflammatory pain model. The effect of vehicle (VEH, 5% PEG/5% Tween-80 in saline, 10 mL/kg, i.p.) and celastrol (CEL, 0.3 mg/kg, i.p.) on carrageenan-induced edema (**A**) and pain hypersensitivity (**B**). *** *p*< 0.001 *vs.* non-carrageenan injection (CT), ## *p* < 0.01 *vs.* VEH; one-way ANOVA followed by Bonferroni’s multiple comparison test, *n* = 5–6. The time- and dose-dependent effect of celastrol on carrageenan-induced edema (**C**) and pain hypersensitivity (**D**). * *p* < 0.05, ** *p* < 0.01, *** *p* < 0.001 *vs.* vehicle, two-way ANOVA with Bonferroni’s post-tests, *n* = 5–6/group.

To investigate the effects of celastrol on inflammation and pain, we used a carrageenan-induced inflammatory pain model to test the pain behavior in response to celastrol administration. First, inflammatory pain was induced by left paw intraplantar injection (i.pl.) of carrageenan, and inflammatory pain was evaluated by the induction of local edema and the rapid mechanical allodynia test [26] 6 h after carrageenan injection. Compared with the right paw controls, left paws of mice exhibited local edema (Figure 1A) and a decrease of the withdraw threshold in the allodynia test (Figure 1B). When we pretreated mice with 0.3 mg/kg of celastrol (i.p.) 30 min before carrageenan administration, we found that celastrol significantly reduced the paw edema (*p* < 0.01, *n* = 5–6) and the mechanical hyperalgesia (*p* < 0.01, *n* = 5–6) (Figure 1A,B) induced by carrageenan injection. In addition, we gave various doses of celastrol (0.1–1 mg/kg, i.p.) and analyzed pain-related parameters at 2, 4, 8, 24 and 48 h after carrageenan injection. Compared to vehicle (5% Tween 80/5% PEG/saline, 10 mL/kg, i.p.), celastrol administration produced a dose-dependent inhibition on local edema and hyperalgesia in carrageenan mice. Notably, while the inhibition effects of celastrol on local edema and hyperalgesia were observed up to 48 h with a high dose of celastrol (1 mg/kg, i.p.), the greatest reduction of edema and pain occurred between 4 and 8 h and between 2 and 4 h, respectively, after celastrol administration of all given doses (Figure 1C). The allodynia test showed that rapid mechanical hyperalgesia was developed 2 h after carrageenan injection, and the effects of celastrol on analgesia exhibited a dose-dependent and a time-dependent effect (Figure 1D). Together, our data demonstrated the profound anti-inflammatory and antinociceptive effects of celastrol on a carrageenan-induced inflammatory pain model.

### 2.2. Celastrol Produced an Antinociceptive Effect through the Cannabinoid Receptor-2 (CB_2_) Signal in Carrageenan-Induced Inflammatory Pain

**Figure 2:**
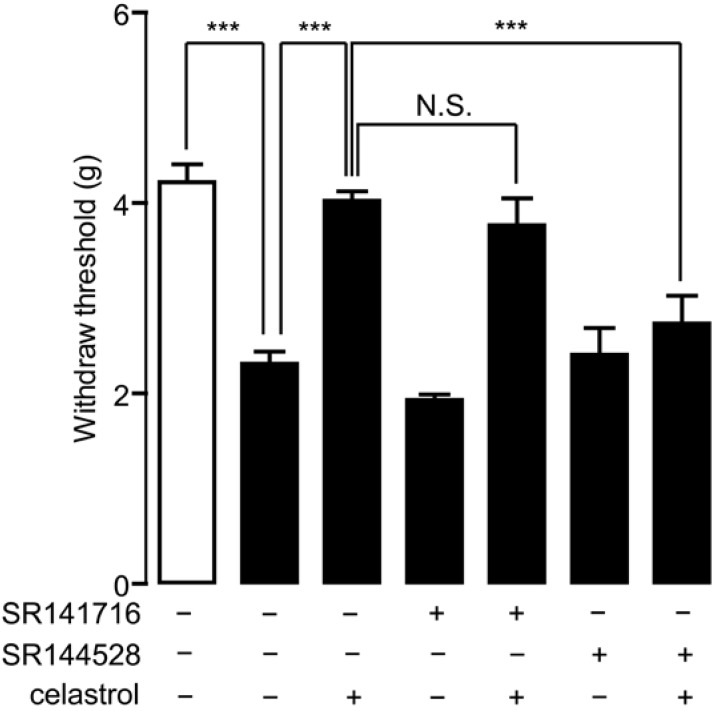
Analgesia effect of celastrol in carrageenan-induced inflammatory pain was mediated by cannabinoid receptor-2 (CB_2_) signaling. The effects of celastrol, SR141716, SR144528, a combination of celastrol and SR141716 and a combination of celastrol and SR144528 on mechanical hyperalgesia, assessed by the allodynia test, in carrageenan-injected mice (closed bar). Open bar, non-carrageenan injected mice; vehicle, 5% PEG/5% Tween-80 in saline, 10 mL/kg, i.p; celastrol, 0.3 mg/kg, i.p.; SR141716, 1 mg/kg, i.p.; SR144528, 1 mg/kg, i.p. *** *p* < 0.001; N.S., not significant; one-way ANOVA followed by Bonferroni’s multiple comparison test, *n* = 8.

The interaction between celastrol and the endocannabinoid system has been demonstrated previously [20,24]. Celastrol inhibited the activity of MGL, an enzyme deactivating 2-AG [24], which reduced inflammatory nociception mediated by cannabinoid signals [20]. To investigate whether the cannabinoid system involves in the analgesia property of celastrol, we blocked cannabinoid signals with CB_1_ or CB_2_ antagonists and assessed the analgesia effect of celastrol in carrageenan-induced inflammatory pain mice. Mice were pre-treated with selective CB_1_ antagonist SR141716 (1 mg/kg, i.p.) or CB_2_ antagonist SR144528 (1 mg/kg, i.p) 15 min before celastrol (0.3 mg/kg, i.p) regimen and then examined for pain withdraw threshold by allodynia test 2 h after carrageenan injection. We found that the analgesia effects of celastrol on carrageenan-induced pain were eliminated by SR144528 (Figure 2), but not by SR141716, suggesting that CB_2_ signaling participated in the antinociceptive effect of celastrol in carrageenan-induced inflammatory pain.

### 2.3. Celastrol Suppressed the mRNA Expression Levels of Inflammatory Cytokines Induced in Carrageenan-Injected Mice

**Figure 3:**
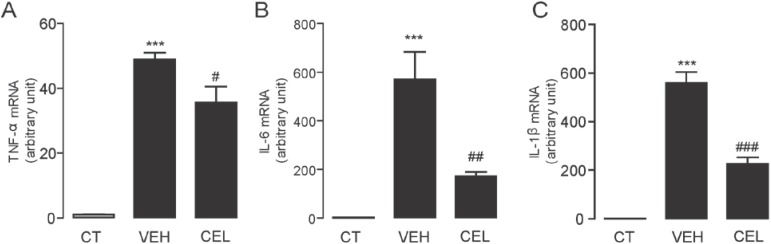
Celastrol suppressed carrageenan-induced inflammation. The effect of vehicle (VEH, 5% PEG/5% Tween-80 in saline, 10 mL/kg, i.p.) and celastrol (CEL, 0.3 mg/kg, 10 mL/kg, i.p.) on the mRNA expressions of (**A**) TNF-α; (**B**) IL-6; and (**C**) IL-1β in the paw tissues of carrageenan injected mice (close bars). *** *p* < 0.001 *vs.* non-carrageenan injection (CT, open bars); # *p* < 0.05, ## *p* < 0.01, ### *p* < 0.001 *vs.* VEH, one-way ANOVA with Bonferroni’s multiple comparison test. *n* = 6.

The hyperalgesia activity in carrageenan mice has been considered to be associated with the inflammatory process that causes nociceptive sensitization. We further examined the mRNA expression levels of inflammatory cytokines, including TNF-α, IL-6 and IL-1β, in the paws of mice with or without carrageenan injection by quantitative RT-PCR analysis. Consistent with other reports [26], carrageenan dramatically increased mRNA expression levels of TNF-α, IL-6 and IL-1β in left paw tissues, which were injected with carrageenan, compared to those in the right paw tissues; the controls injected with saline (Figure 3). Administration of celastrol (0.3 mg/kg, i.p) significantly suppressed mRNA expression levels of TNF-α (VEH, 48.98 ± 2.05 *vs.* CEL, 35.58 ± 5.01; *p* < 0.05, *n* = 6), IL-6 (VEH, 570.18 ± 113.30 *vs.* CEL, 171.77 ± 17.27; *p* < 0.01, *n* = 6), and IL-1β (VEH, 560.65 ± 44.44 *vs.* CEL, 226.96 ± 27.19; *p* < 0.001, *n* = 6) induced in carrageenan-injected mice.

### 2.4. Celastrol Alleviated Hyperalgesia in Neuropathic Pain Mice

**Figure 4:**
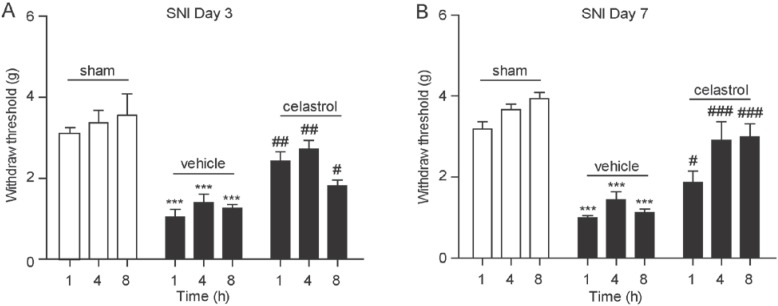
Celastrol reduced hyperalgesia in SNI mice. The time-course effect of vehicle (5% PEG/5% Tween-80 in saline, 10 mL/kg, i.p.) and celastrol (1 mg/kg, 10 mL/kg, i.p.) on mechanical allodynia in SNI mice (closed bars) on the third day post-surgery (**A**); and the seventh day post-surgery (**B**). Sham, sham-operated mice (open bars); 1, 1 h after drug administration; 4, 4 h after drug administration; 8, 8 h after drug administration. *** *p* < 0.001 *vs.* Sham-operated mice; # *p* < 0.05, ## *p* < 0.01, ### *p* < 0.001 *vs.* vehicle, one-way ANOVA with Bonferroni’s multiple comparison test, *n* = 7–8.

In addition to the carrageenan-induced inflammatory pain model, there is increasing evidence suggesting that cannabinoid signals also play an important role in neuropathic pain [27]. Therefore, we further evaluated the analgesia effect of celastrol in the spared nerve injury (SNI) neuropathic pain model. Consistent with previous reports that the ligation of tibial and common peroneal nerves induced chronic neuropathic pain [28], we detected a significant decrease of mechanical withdraw thresholds in SNI mice by the allodynia test, compared with sham-operated mice on the third day post-surgery (Figure 4A) and the seventh day post-surgery (Figure 4B). On the third day post-surgery and the seventh day post-surgery SNI mice, we conducted an allodynia test 1, 4 and 8 h after the administration of celastrol (1 mg/kg, i.p.). Figure 3 shows that celastrol significantly increased the pain withdraw threshold response to mechanical stimuli on the third day post-surgery SNI mice (Figure 4A) and the seventh day post-surgery SNI mice (Figure 4B), and this effect lasted up to 8 h after the drug regimen.

### 2.5. Analgesia Effect of Celastrol in Neuropathic Pain Was Mediated by the CB2 Signal

**Figure 5:**
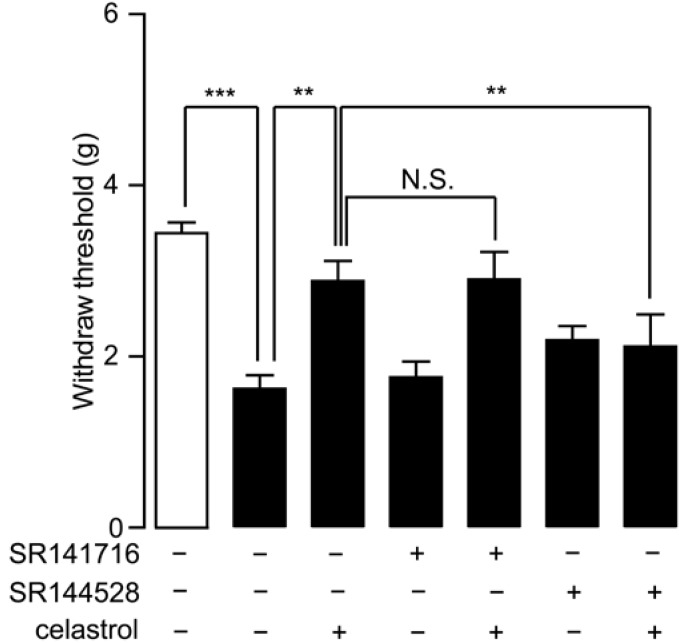
Analgesia effect of celastrol in SNI mice was blocked by the CB_2_ antagonist. The effects of vehicle (5% PEG/5% Tween-80 in saline, 10 mL/kg, i.p.), SR141716 (1 mg/kg, i.p.), SR144528 (1 mg/kg, i.p.) and celastrol (0.3 mg/kg, i.p.) on the mechanical allodynia test in the seventh day post-surgery SNI mice (closed bars). Open bars, sham-operated mice. Pain withdraw thresholds were measured two hours after drug administration. N.S., not significant; *** *p* < 0.001, ** *p* < 0.01, one-way ANOVA with Bonferroni’s multiple comparison test, *n* = 5–6.

Cannabinoid signals have played an important role in various types of neuropathic pain [29,30,31]. Here, we further investigated whether cannabinoid signals were involved in the analgesia effect of celastrol on the SNI neuropathic pain model. We used pharmacological blockers of cannabinoid signals, including SR141716 and SR144528, to examine mechanical allodynia on the seventh day post-surgery SNI mice. Compared to sham-operated mice, SNI mice revealed a hypernociceptive response to mechanical stimuli and a single injection of either SR141716 or SR144528 on the seventh day post-surgery SNI mice had no effect on nociception (Figure 5). On the seventh day post-surgery SNI mice, celastrol (1 mg/kg, i.p.) significantly attenuated hyperalgesia (CEL, 2.88 ± 0.24 *vs*. VEH, 1.62 ± 0.16, *p* < 0.01, *n* = 5–6), and the analgesia effect of celastrol was eliminated by the selective CB_2_ antagonist, SR144528, but not by the selective CB_1_ antagonist, SR141716 (Figure 5).

## 3. Experimental Section

### 3.1. Drug Administration

In the carrageenan-induced inflammatory pain model, mice were treated with various doses of celastrol (0.1, 0.3 or 1 mg/kg (10 mL/kg, i.p.)) or vehicle (5% Tween 80, 5% PEG 400 in saline solution, 10 mL/kg, i.p.) 30 min before carrageenan injection. In the SNI neuropathic pain model, mice received celastrol (0.3 mg/kg, 10 mL/kg, i.p.) or vehicle (5% Tween 80, 5% PEG 400 in saline solution, 10 mL/kg, i.p.) on the third day post-surgery and the seventh day post-surgery. To evaluate the role of CB signaling on the celastrol effect, SR141716 (1 mg/kg, i.p.) or SR144528 (1 mg/kg, i.p.) was given 15 min before the administration of celastrol.

### 3.2. Carrageenan-Induced Inflammatory Pain

Inflammatory pain was induced by injecting λ-carrageenan (2% in sterile saline, 20 μL) subcutaneously into the left hind paw using a 27-gauge needle (Gaoge, Shanghai, China). Edema and an allodynia test were conducted at 2, 4, 6, 8, 24 and 48 h after carrageenan administration.

### 3.3. Spared Nerve Injury (SNI) Surgery

The procedure was performed as previously reported [32] with slight modification. Briefly, C57BL/6J mice were anesthetized with pentobarbital sodium (80 mg/kg, i.p.) (Sangon Biotech, Shanghai, China), and the hair in the operative area was shaved from slightly below the knee area to the hip area of the left thigh. After disinfection with 75% ethanol, a 1-cm incision on the left thigh was made in the longitudinal direction proximal to the knee, and muscle layers were separated by blunt dissection; the biceps femoris muscle was identified. The mouse was placed under a stereomicroscope, and the sciatic nerve was visualized. Three terminal branches of the sciatic nerve, the sural nerve, common peroneal nerve and tibial nerve, were identified. We used a pair of tweezers to separate nerves, placed a suture (6-0 suture) under the two branches, the tibial nerve and common peroneal nerve, made a tight surgical knot, gently grabbed the nerves with the suture and cut the nerves with a micro-scissor. The scission was closed with a suture, cleaned with 70% ethanol and covered with sterilized gauze pad. This procedure made the ligation of the tibial nerve and common peroneal nerve, but kept the sural nerve intact. Sham-operated controls underwent the same procedure without nerve lesion. After surgery, mice were placed in a clean cage with a heating pad and ensured easy access to water and chow.

### 3.4. Allodynia Test

Pain nociception was determined using the automated Dynamic Plantar Aesthesiometer system (Ugo Basile, Comerio, Italy). Briefly, mice were placed into the enclosure compartments with a wire grid bottom, a total 12 units, at least 30 min prior to the experiment, to be habituated to the environment. A touch stimulator was located under the target area of the mouse paw; using the adjustable angled mirror, a thin steel filament (diameter 0.5 mm) was pushed against the plantar surface of mouse paw with increasing force, until the paw withdrawal, and the force was recorded automatically. The apparatus was set from 0 g to a maximum of 5 g within 20 s (ramp 0.25 g/s) and manually stopped when the force reached the maximum. The paw withdrawal threshold was calculated as the mean of 5 consecutive assessments at intervals of 20 s.

### 3.5. Edema Measurement

Paw edema was measured by the changes in paw volume by water displacement with a plethysmometer (Ugo Basile, Comerio, Italy). The plethysmometer contained two vertical water-filled interconnecting Perspex tubes; the large one (A, 18-mm diameter) was used to measure fluid displaced by the paw, and the small tube contained a transducer with data analysis software (Ugo Basile, Comerio, Italy). The edema was calculated by subtracting the volume of the contralateral paw from that of carrageenan-injected paw.

### 3.6. Quantitative Real-Time PCR

Total RNA was extracted from mice paw tissue with TRIzol^®^ (Invitrogen, Shanghai, China), and the complementary DNA was synthesized using ReverTra Ace qPCR RT Kit (TOYOBO, Shanghai, China) following the manufacturer’s instructions. Real-time quantitative PCR was performed on a 7500 Real Time PCR System (Applied Biosystems, Shanghai, China) using a SYBR Premix Ex TaqTM II (Tli RNaseH Plus) kit (TaKaRa, Dalian, China). The expressions of target genes were normalized with the internal control gene, glyceraldehyde 3-phosphate dehydrogenase (GAPDH), and relative changes of gene expression were calculated using the 2^−ΔΔ*C*t^ method. The primers for mouse genes were synthesized by Sangon Biotech (Shanghai, China), and the sequences were as follows: (i) TNF-α, F: AATGGCCTCCCTCTCATCAGTTCT, R: TGAGATAGCAAATCGGCTGACGGT; (ii) IL-6, F: AATTAAGCCTCCGACTTGTGAAG, R: CTTCCATCCAGTTGCCTTCTTG; and (iii) IL-1β, F: TGTAATGAAAGACGGCACACC, R: TCTTCTTTGGGTATTGCTTGG; (iiii) GAPDH, F: TTGCTGTTGAAGTCGCAGGAG; R: TGTGTCCGTCGTGGATCTGA.

### 3.7. Statistical Analysis

Results are presented as the means ± standard error (SEM). Differences between groups were analyzed with multiple variances (one-way ANOVA or two-way ANOVA), followed by Bonferroni’s test using GraphPad Prism 5 software (GraphPad Software, San Diego, CA, USA). Statistical differences were considered significant at *p* < 0.05.

## 4. Conclusions

Altogether, our data demonstrated that celastrol significantly reduced nociceptive pain and elucidated the possible mechanism underlying the analgesia effect of celastrol through CB_2_ signaling. The cannabinoid-mediated analgesia effect has been fully defined for many years; however, the undesired central nervous system adverse effects limited its use in humans [33]. As increasing evidence supporting the utilization of selective CB_2_ agonists for managing pain [34], our findings showed that the analgesia effects of celastrol were mediated by CB_2_ signaling. The pharmacological action of celastrol on pain is concomitant with the following: (i) as a potent MGL inhibitor, celastrol activates CB_2_ signaling through an increase of CB_2_ endogenous ligand 2-AG, a substrate catalyzed by MGL; and (ii) the blockage of CB_2_ signaling by selective antagonist completely eliminates celastrol’s pharmacological effects. Without exploring the target site of celastrol’s action in animal models, we lack the information regarding the endogenous 2-AG levels, and this limitation will be studied in the future. In addition, investigating whether celastrol directly interacts with the CB_2_ receptor will be of great interest and value for the full elucidation of celastrol’s pharmacological properties. As there are few drugs effectively managing neuropathic pain and their common side effects restrict their use in certain patients [35], our findings might usher in a new field for the development of safe and efficacious drugs for chronic pain.
